# Integration of Data and Predictive Models for the Evaluation of Air Quality and Noise in Urban Environments

**DOI:** 10.3390/s24020311

**Published:** 2024-01-05

**Authors:** Jaime Govea, Walter Gaibor-Naranjo, Santiago Sanchez-Viteri, William Villegas-Ch

**Affiliations:** 1 Escuela de Ingeniería en Ciberseguridad, Faculatad de Ingenierías y Ciencias Aplicadas, Universidad de Las Américas, Quito 170125, Ecuador; jaimealejandro.govea@udla.edu.ec; 2 Carrera de Ciencias de la Computación, Universidad Politécnica Salesiana, Quito 170105, Ecuador; wgaibor@ups.edu.ec; 3 Departamento de Sistemas, Universidad Internacional del Ecuador, Quito 170411, Ecuador; ssanchez@uide.edu.ec

**Keywords:** air quality, urban noise, urban planification

## Abstract

This work addresses assessing air quality and noise in urban environments by integrating predictive models and Internet of Things technologies. For this, a model generated heat maps for PM2.5 and noise levels, incorporating traffic data from open sources for precise contextualization. This approach reveals significant correlations between high pollutant/noise concentrations and their proximity to industrial zones and traffic routes. The predictive models, including convolutional neural networks and decision trees, demonstrated high accuracy in predicting pollution and noise levels, with correlation values such as R2 of 0.93 for PM2.5 and 0.90 for noise. These findings highlight the need to address environmental issues in urban planning comprehensively. Furthermore, the study suggests policies based on the quantitative results, such as implementing low-emission zones and promoting green spaces, to improve urban environmental management. This analysis offers a significant contribution to scientific understanding and practical applicability in the planning and management of urban environments, emphasizing the relevance of an integrated and data-driven approach to inform effective policy decisions in urban environmental management.

## 1. Introduction

Air quality in urban environments has become critical in public health and urban planning. Air quality measurement and modeling have historically relied on ground-based monitoring networks, which provide valuable data but are often limited in geographic scope. The rapid growth of urban areas has given rise to environmental and quality-of-life challenges. The most pressing problems are air quality and noise pollution. Air pollution, characterized by the presence of fine particles (PM2.5), volatile organic compounds (VOCs), and nitrogen oxides (NOx), among other pollutants, has harmful effects on human health and the environment [1,2]. On the other hand, noise pollution, measured in terms of noise levels, can also negatively affect the health and well-being of people living in urban environments [3,4].

Concern about these problems has led to the implementation of regulations and policies to control polluting emissions and reduce city noise levels [5]. However, the complexity of these environmental challenges requires more comprehensive approaches to understanding and addressing their interactions [6,7]. Recent studies have integrated satellite approaches to overcome these limitations, offering a broader perspective on air pollution [8]. However, these methods face challenges in capturing urban pollutants’ detailed temporal and spatial dynamics [9].

Despite advances in satellite technology and modeling, there remains a significant gap in the comprehensive understanding of urban air quality. Although accurate under controlled conditions, laboratory studies often do not reflect the complexity and variability of natural urban environments [10]. Furthermore, current predictive models, although practical, usually do not effectively integrate the diversity of pollution sources and their interaction with dynamic urban factors [11].

Furthermore, incorporating emerging technologies, such as the Internet of Things (IoT), has opened new possibilities to dynamically and in real-time monitor and model air quality. These advances represent a shift toward a more holistic approach, combining data from multiple sources and technologies for a complete understanding of urban pollution patterns [12]. However, adopting these technologies also introduces new challenges, such as effectively integrating and analyzing large volumes of data and the precise calibration of a sensor network.

This work comprehensively addresses air quality and noise in urban environments. This proposal is based on two fundamental pillars: multidisciplinary environmental data and traffic data [13]. This work uses the appropriate technology to measure and record these data continuously. This includes air quality sensors that monitor PM2.5, VOCs, and NOx, as well as noise sensors that record noise levels at different locations in the city [14,15]. Vehicle traffic is one of the primary sources of air and noise pollution in cities.

Integrating these pillars provides a holistic view of air quality and noise in the city. It also allows the identification of critical areas that require attention and specific mitigation measures [16,17]. Predictive models, such as convolutional neural networks (CNNs) and decision trees, are used to develop a robust predictive framework. This allows us to understand the current situation and foresee how these problems could evolve in the future under different urban scenarios [18,19].

The results of this work support the negative influence of urban sources, such as industrial areas and heavy traffic routes, on air quality and noise. This highlights the importance of addressing these issues comprehensively and the need to implement specific mitigation strategies in critical areas. Additionally, a significant correlation was identified between air quality and noise levels in the monitored areas of the city. This underlines the importance of addressing these problems jointly in urban planning [20]. The information provided by our research is essential to make informed and strategic decisions in managing air quality and noise in urban environments, contributing to healthier and more sustainable cities.

## 2. Materials and Methods

The generation of environmental and urban data in the city is used to develop the method. In addition, information on industrial areas and traffic routes, the generation of PM2.5 and noise heat maps, and the integration of environmental and traffic data are included. This information allows the evaluation of predictive models for air quality and noise levels, highlighting the performance metrics used.

### 2.1. Review of Similar Works

Numerous studies have addressed the topic from various perspectives in urban environmental monitoring, using multiple methodologies and technologies. Reviewing the existing literature, we found that a common approach involves using IoT sensor networks to collect data on factors such as air quality and noise in urban environments. These studies have been fundamental to understanding how pollution and other environmental factors affect city life [21].

For example, some studies have focused on deploying sensor networks to precisely monitor levels of pollutants such as PM2.5 and NOx, providing valuable real-time air quality data. These projects have demonstrated the effectiveness of using low-cost, easily implemented technology to obtain critical environmental data [22,23]. Additionally, other work has explored the use of advanced data processing algorithms to interpret large environmental data sets, allowing researchers and policymakers to gain a deeper understanding of ecological patterns and trends [23].

This work builds on these previous studies and seeks to advance the field by integrating more advanced IoT technologies and developing sophisticated algorithms for data analysis. Unlike previous studies that might have focused on specific aspects of environmental monitoring, this proposal aims to provide a holistic and systematic approach [24]. This includes not only the collection of environmental data but also its detailed analysis to inform urban management policies and strategies better [25,26].

The contribution of this work to the phenomenon under study not only expands the scope of the data collected, but also improves the accuracy and usefulness of the analysis of this data. In doing so, we hope to offer new perspectives and solutions to urban environmental challenges, thereby creating healthier and more sustainable cities [27,28]. Additionally, our focus on optimizing and automating data collection and analysis represents a significant advance in the efficiency and effectiveness of urban environmental monitoring.

### 2.2. Monitoring Platform Design

Open data available on the web from recognized sources, such as the Urban Data Platform of the European Commission, France’s National Address Base, and the Open Data Barometer, are used to design the urban environmental monitoring platform. These sources offer valuable and updated information on urban variables, integrated into the platform developed to enrich the analysis and understanding of city environmental challenges.

#### 2.2.1. Platform Architecture

**Figure 1:**
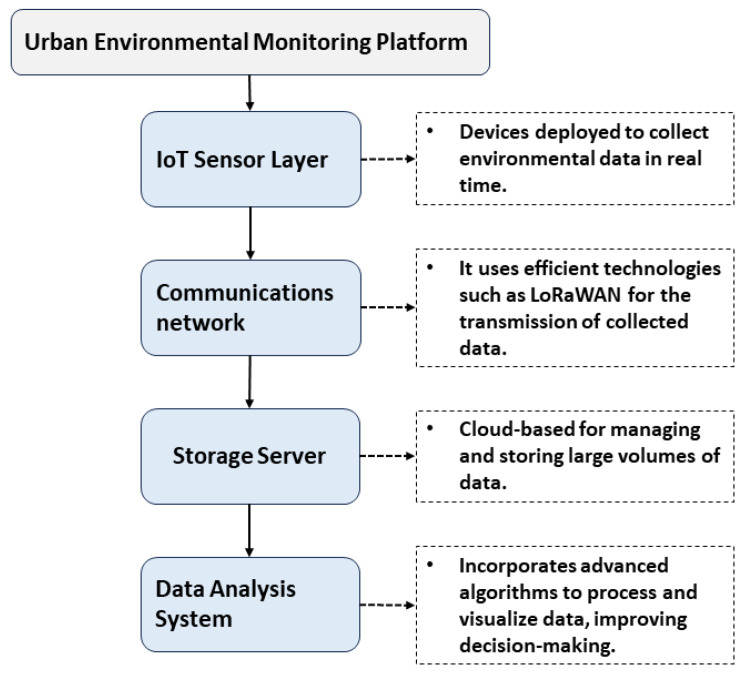
Block diagram of the urban environmental monitoring platform.

Figure 1 shows the block diagram of the urban environmental monitoring platform, detailing its structure, composed of four main layers: the IoT Sensor Layer, the Communications Network, the Storage Server, and the Data Analysis System [29,30]. The diagram illustrates the structure and data flow of our urban environmental monitoring platform.

The sensor layer is responsible for collecting environmental data in real-time. These data are transmitted over a network that uses efficient technologies such as LoRaWAN [31,32]. The cloud-based storage server manages and stores large volumes of collected data. The data analysis system processes and interprets this information using advanced algorithms, which improves decision-making related to the urban environment.

#### 2.2.2. Selection of IoT Technologies

For implementing the urban environmental monitoring platform, a range of IoT technologies has been carefully selected based on their efficiency, accuracy, and reliability. The air quality sensors will be optical for PM2.5 and PM10 particles and electrochemical for gases such as NOx and SOx, providing essential data on atmospheric pollution [33]. The infrared sensors will measure the levels of CO and CO_2_, while the specific devices for ozone will give us information about this gas critical for public health and environmental quality [34].

Regarding noise pollution, calibrated microphones will offer us precise measurements of noise levels, allowing us to address this omnipresent urban pollutant effectively [35]. For data communication, technologies such as LoRaWAN and NB-IoT, ideal for low-power, long-range IoT data transmission, and networks for applications requiring real-time transmission and support for a high density of connected devices, were chosen [36,37,38].

The cloud infrastructure, selected for its scalability and robustness, serves as the core for data storage and processing, using big data tools to manage and analyze the large volumes of information collected [39,40]. This is complemented by data visualization software and machine learning algorithms to interpret the data and generate predictive models that inform and improve urban environmental planning and response decisions. Each of these components ensures maximum operational consistency and energy efficiency, thus ensuring high-quality data collection and meaningful insights.

In selecting IoT technologies for our study, in addition to efficiency, accuracy, and reliability, the specific suitability of each sensor for complex urban environments was considered. Sensor calibration was performed using recognized standards in controlled environments to ensure accuracy in detecting contaminants and noise levels. This included exposing the sensors to known pollutant concentrations and decibels in a range that reflects actual urban conditions.

The sensor placement and deployment strategy were determined to capture a complete pollution and noise profile. The sensors were placed at strategic points, such as high-traffic intersections, residential and commercial areas, and near industrial emission sources, providing a comprehensive and detailed perspective of urban environmental conditions.

For validation, data collected by the sensors were compared to reference measurements obtained using conventional methods. This cross-validation was carried out in multiple locations and different environmental conditions, thus guaranteeing the reliability of the sensors in various urban situations.

Additionally, a regular maintenance and recalibration protocol was implemented for the sensors, considering factors such as sensor degradation over time and significant environmental changes, ensuring data consistency and accuracy over time.

Integrating these data into the urban environmental monitoring platform, combined with cloud infrastructure, big data, and machine learning algorithms, ensures high-quality data collection, and facilitates its analysis, model generation, and accurate predictions.

### 2.3. Data Collection and Processing

The study was conducted in a simulated city representative of a typical urban environment, with clearly defined residential, commercial, and industrial areas. To obtain a diverse and representative sample of environmental conditions, 150 IoT sensors were deployed in multiple strategic locations. Data from the sensors distributed in these areas were collected and analyzed over 6 months, providing data on air quality and noise levels. This approach allowed us to capture a detailed image of environmental patterns in a complex urban context.

Data collection and processing is based on integrating multiple open data sources and IoT instrumentation deployed throughout the urban environment. Data from the European Commission’s Urban Data Platform, the French national address database, and the Open Data Barometer enrich our analysis with contextual and comparative information.

Specifically, selected IoT sensors collect environmental data such as particle concentrations and noise levels. These data are quantitative, with volumes anticipated to be considerable, given the granularity and frequency of measurements required for detailed analysis. For example, particle sensors could generate up to 10 GB of data weekly, updating every hour, while microphones could generate around 2 GB of data with updates every half hour.

In data acquisition, a differentiated sampling frequency for each type of sensor is established and optimized to capture relevant short- and long-term environmental variations. This varied frequency allows for detailed, real-time air quality and noise analysis in different urban areas. The geographic coverage of the sensors covers a diversity of urban areas, from residential to industrial and commercial areas, thus ensuring the collection of data representative of the city. Additionally, real-time filtering and verification protocols are implemented to ensure the quality and accuracy of the data collected. This included data normalization to ensure consistency between different sensors and cross-validation with standard measurement methods, strengthening the reliability of the data set.

Processing these data begins with a cleaning phase to correct or remove outlier reads, followed by normalization to allow meaningful comparisons between data sets and locations. The processed data will be stored in a centralized repository, where big data techniques and machine learning algorithms will process the information to identify trends, patterns, and correlations. Table 1 shows a summary of the data used.

Open data complement the data collected by sensors, providing a broader context for interpreting the data and helping to validate the prediction and analysis models developed. The processing methodology will be designed to be scalable and adaptable to adjust to emerging needs and the evolution of data collection technologies.

This work effectively integrated traffic data and other relevant urban elements with environmental data collected by sensors. Tools such as geographic information systems and extensive data analysis platforms such as Hadoop and Spark are used in data integration. These tools allow for a compelling fusion of traffic data and other urban indicators with environmental data. We use parallel processing algorithms to efficiently handle the volume and complexity of data efficiently, ensuring accurate and detailed integration. This multifaceted approach allows for developing more effective and sustainable urban planning strategies.

The representativeness of the collected data is carefully evaluated to ensure that they accurately reflect actual urban conditions. Variations in urban distribution, population density, and industrial activity are considered to ensure the generalizability of the results. However, it is essential to recognize the limitations inherent in using IoT sensors and simulation models. These include potential biases in sensor placement and constraints on representing the full complexity of the urban environment. These factors were critically analyzed to understand their impact on the study’s conclusions and formulate recommendations for future research and practical applications.

Effective management of data collected through IoT sensors is crucial to this work. With the use of Hadoop and Spark, a scalable data storage system was implemented to manage the vast amount of information collected efficiently. These platforms enabled fast and secure processing, which is essential for real-time analysis. Big data techniques were applied to analyze these data, including machine learning algorithms and statistical analysis. This approach allowed us to extract meaningful patterns and correlations from the data, which was unattainable with traditional methods due to the complexity and magnitude of the data. However, several challenges were faced, such as data integrity and processing efficiency. To overcome these issues, we established rigorous data verification and filtering protocols and processing optimizations to improve the speed and accuracy of analysis.

The use of IoT technologies for environmental monitoring involves facing various challenges. One of the main obstacles was the accurate calibration of the sensors. For example, PM2.5 sensors required periodic calibrations to counteract the drift caused by environmental factors such as humidity and temperature. We implement regular calibration protocols and compare data to reference sensors to ensure accuracy.

Data integrity was another significant challenge. Noise sensors were subject to external interference that could affect accuracy. We use data filters and statistical analysis techniques to address this to identify and correct potential errors.

Additionally, network reliability is crucial for effective data transmission. Network redundancy systems and local data storage mechanisms were established to ensure continuity in data collection and avoid data loss due to fluctuations in network connectivity.

Table 2 summarizes the key challenges faced in calibration and data collection with different types of IoT sensors and the strategies implemented to address these issues. The table provides a comprehensive view of how the accuracy and integrity of the data were ensured, highlighting both the technical obstacles and the solutions applied to overcome them in the context of urban environmental monitoring.

### 2.4. Data Analysis and Algorithm Development

Data analysis and algorithm development are essential for turning large volumes of raw data into actionable insights. The algorithms to be developed cover several areas of data analysis:

Preprocessing of the collected data is carried out in several stages. Initially, the data are subjected to cleaning that involves the elimination of outliers using statistical methods such as the Tukey test or the analysis of standard deviations. Missing values are treated using imputation techniques, such as mean imputation or k-nearest neighbors’ imputation, depending on the nature of the data [41]. Normalization is applied to the data to homogenize the scale from different sources, using min–max normalization or Z-score standardization methods.

For descriptive statistical analysis, measures of central tendency and dispersion are used, and for exploratory data analysis (EDA), visual techniques such as histograms, boxplots, and scatterplots are applied. Regression algorithms, classifiers such as support vector machines, and neural networks are used to predict pollution levels in predictive modeling [42,43]. Clustering algorithms identify patterns in unlabeled data, such as k-means or Density-Based Spatial Clustering of Applications with Noise (DBSCAN).

The interpretation of the data is carried out through analyzing the outputs of these models. At the same time, the visualization is facilitated through interactive dashboards that allow users to explore the data using filters and controls. Time series are visualized through line or area graphs. Each algorithm and visualization technique is selected and customized to the specific needs of the analysis, ensuring that results are both technically sound and accessible to end users, including decision-makers and the public.

Regarding the architecture of the CNNs, a model with multiple layers was used, including convolutional layers for feature extraction, pooling layers for dimensionality reduction, and finally, fully connected layers for classification. ReLU activation functions were used for the convolutional layers and Softmax for the output layer. A maximum tree depth was defined for decision trees, and entropy criteria were utilized for node splitting.

The CNN architecture consisted of three convolutional layers, each followed by a pooling layer to reduce dimensionality. The convolutional layers had 32, 64, and 128 filters, respectively, with a kernel size 3 × 3. Max pooling was used for the reduction layers. The network ended up with two fully connected layers of 64 and 32 nodes.

A maximum depth of 10 levels was set for the decision trees, and the Gini impurity criterion was used for splits. Parameter tuning was performed with a grid search, evaluating combinations of tree depth and number of leaf nodes. Five-fold cross-validation was used to avoid overfitting and ensure the model’s generalization.

Parameter tuning was performed using cross-validation and grid search techniques to find the optimal combination of hyperparameters. This approach ensured the generalization and effectiveness of the models. Validation and testing were carried out on separate data sets, using metrics such as accuracy, sensitivity, and specificity to evaluate model performance.

### 2.5. Implementation and Testing

Pilot tests and simulations of the proposed platform are carried out in different phases.

#### 2.5.1. Pilot Tests in Urban Environments

The implementation process of the urban environmental monitoring platform begins with selecting metropolitan areas with high population and traffic density, focusing on regions with diverse environmental and topographic conditions. This will include both educational and residential areas as well as industrial areas. Next, we deploy IoT sensors that measure air quality and noise at strategic points, such as busy intersections, parks, and proximity to industrial emission sources [44].

Once installed, the sensors continuously transmit data to the processing center. These data include levels of air pollutants, noise decibels, and relevant meteorological parameters. Ongoing monitoring and maintenance of these sensors will be essential to ensure the integrity and accuracy of the data collected, which involves performing regular calibrations and upkeep of the devices. Subsequently, we will use the collected data to evaluate both the sensors’ efficiency and the analysis algorithms’ precision. This will include comparisons with already established environmental monitoring stations and a detailed correlation analysis between the different types of data collected [45]. Based on the results obtained from these evaluations, we will make the necessary adjustments to both the sensor network configuration and the data processing algorithms to improve the overall accuracy and efficiency of the platform.

#### 2.5.2. Computational Simulations

Mathematical models are used to simulate different environmental scenarios. For example, for the dispersion of pollutants, the advection–diffusion equation is applied:

(1) $$C\left(x,t\right)=\frac{Q}{{\left(4\pi Dt\right)}^{1/2}}\;exp\left(-\frac{{\left(x-ut\right)}^{2}}{4Dt}\right)$$

where *C*(*x*, *t*) is the concentration of the pollutant at time *t* and position *x*, *Q* is the emission source, *D* is the diffusion coefficient, *u* is the wind speed, and *x* is the distance from the source.

#### 2.5.3. Performance and Effectiveness Evaluation

For classification algorithms, accuracy, sensitivity (true positive rate), and specificity (true negative rate) are evaluated using the confusion matrix. In addition, cross-validation techniques, such as k-fold, are applied to validate the robustness of the predictive models. To validate the accuracy of the algorithms, the model predictions are compared with historical data and accurate observations. The determination coefficients R2 are used to quantify how much of the variability in the observed data is explained by the model. To evaluate the operation of the model, stress tests are carried out on the platform to guarantee its operation under extreme conditions, such as high pollution levels or adverse weather events.

The methods used to calculate the accuracy, sensitivity, and specificity of the algorithm in detecting sabotage are:

- *Precision*: This metric evaluates the number of true positives (correctly identified sabotages) relative to all identified positives (correct and incorrect).
  (2) $$Precision=\frac{True\;Positives}{True\;Positives+False\;positives}$$
- *Sensitivity (True Positive Rate)*: Measures the proportion of real sabotages the algorithm correctly identifies.
  (3) $$Sensitivity=\frac{True\;Positives}{True\;Positives+False\;Negatives}$$
- *Specificity*: Evaluates the proportion of normal operations that the algorithm correctly identifies; that is, it does not incorrectly mark them as sabotage.
  (4) $$Specificity=\frac{True\;Negatives}{True\;Negatives+False\;positives}$$

These metrics are calculated using the confusion matrix, which compares the algorithm’s predictions with the actual data labels. Accuracy, sensitivity, and specificity provide a comprehensive view of the algorithm’s performance.

## 3. Results

The results of this study reveal statistically significant correlations between high pollution/noise areas and proximity to industrial zones and main roads in the simulated city. A negative influence of urban sources on air quality and noise was observed, highlighting the importance of adequate mitigation measures and urban planning. The predictive models presented high levels of precision, with R2 values of 0.93 for PM2.5 and 0.90 for noise, which supports their usefulness in environmental management. The direct correlation between air quality and noise levels underscores the need to address these issues comprehensively in urban planning.

### 3.1. System Implementation

In the effective implementation of our urban environmental monitoring system. We highlight the importance of accurate configuration and calibration of IoT sensors and communications networks to ensure air quality and noise data collection in urban environments. This implementation is crucial in obtaining relevant and accurate data, essential in analysis and decision-making.

#### 3.1.1. Infrastructure Description

**Figure 2:**
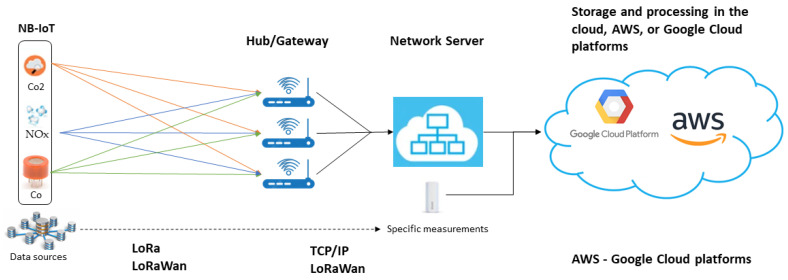
Architecture of the integrated urban environmental monitoring system.

The infrastructure presented in Figure 2 is essential for the implemented urban environmental monitoring system, as it allows for efficient ecological data collection. The use of distributed IoT sensors and network technologies such as LoRaWAN and NB-IoT, along with data processing on cloud platforms such as AWS and Google Cloud, is critical to the accuracy and reliability of the results obtained from the system [46,47]. This configuration ensures that the analyzed data reflects urban environmental conditions in real-time, which is essential for the analyses’ validity.

#### 3.1.2. Implementation Process

**Figure 3:**
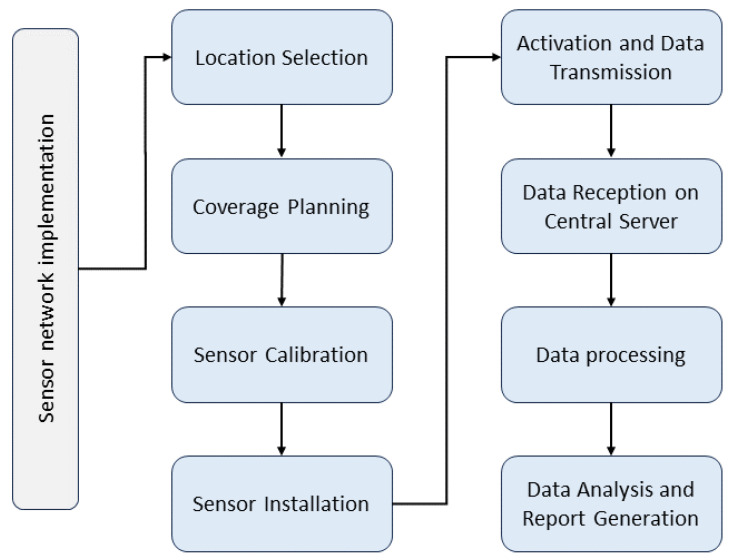
Operational flow for the implementation of the environmental monitoring sensor network.

The sensor network is deployed through a structured sequence that begins with strategically selecting locations, as presented in Figure 3, ensuring optimal and representative city coverage. After installation, each sensor is rigorously calibrated to validate measurement accuracy. The collected data are transmitted to a central server, where it is processed and analyzed, resulting in detailed reports that inform decisions about the urban environment.

### 3.2. Identification and Characterization of Data

The environmental monitoring network collects data for specific pollutants such as PM2.5, PM10, NO_2_, and CO, along with acoustic and meteorological measurements. These data provide an accurate understanding of urban air quality and ambient noise, which is essential for informed environmental management decisions. Regarding data volume, the sensors generate readings at regular time intervals, accumulating significant daily volumes of data. For example, each sensor can generate approximately 1 KB of data per reading, translating to around 1.44 MB per day if data are collected every minute. With sensors deployed, collecting gigabytes of data in a single day is possible.

Table 3 details the volume and characteristics of the environmental data collected by the sensor network. With 150 sensors for PM2.5 and PM10, collecting data every minute with a precision of ±2.5 µg/m^3^, and 100 sensors for NO_2_ and CO, recording every 5 min with a precision of ±2 ppb and ±0.1 ppm, respectively. They generate 1.44 MB and 288 KB of data daily. Noise is measured with an accuracy of ±1 dB, while temperature and humidity are recorded every 10 min with a precision of ±0.5 °C and ±3%, each contributing 144 KB to the daily volume. Fifty sensors collect weather data, providing a detailed basis for deep environmental analysis and predictive modeling.

Statistical techniques were implemented in data preparation to identify and rule out anomalies based on proven methodologies [48,49]. These techniques were selected to ensure that only erroneous or atypical data are removed, preserving the integrity and authenticity of the actual data. Data normalization using the Z-score technique was applied to standardize the data within each contaminant category, allowing for consistent statistical analyses and predictive models without directly comparing different contaminants.

A cleaning phase is initially performed to prepare and segment the data, including eliminating outlier data and imputing missing values. Data normalization is then carried out to ensure compatibility between different data types and sources. Once preprocessed, the data are segmented into training and test sets. Typically, 70–80% of the total data are allocated to training predictive models, while the remaining 20–30% are reserved for the testing and validation phase of the models. These segregated data sets are essential to develop and evaluate the accuracy and generalization of the predictive algorithms applied in the study.

### 3.3. Results of Data Collection and Analysis

**Figure 4:**
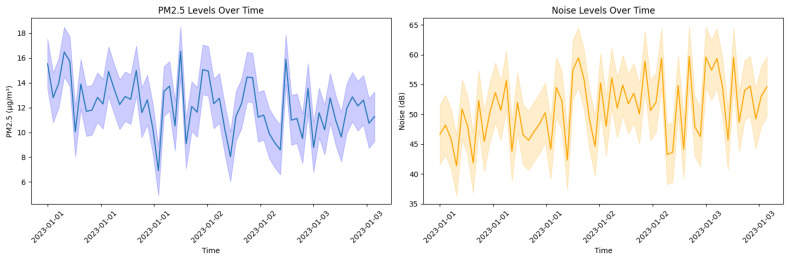
Temporal variations in PM2.5 and urban noise levels.

The results obtained from data analysis allow us to understand the current situation of the urban environment. A dynamic profile of the urban atmosphere has been built by monitoring key parameters such as air quality and noise. Figure 4 shows two graphs representing the temporal variations of two environmental variables of a city for 3 days, starting on 1 January 2023. The first graph shows the concentrations of PM2.5 particles in micrograms per cubic meter, with oscillations reflecting the air quality variability. The horizontal error bars in this graph indicate the precision of the measurements, providing a confidence interval that reflects the possible variation in PM2.5 values due to the inherent uncertainty in data collection. The second graph, which shows ambient noise levels in decibels, also includes error bars that represent the variability and reliability of these measurements. These error bars, calculated from the standard deviation of the collected data, allow us to appreciate the observed trends and evaluate the influence of possible disturbing factors or sporadic events in the urban environment. These graphs, with their corresponding error bars, provide an accurate and transparent representation of the data collected, essential for assessments of the city’s environmental health.

Analysis of the collected data revealed significant trends in urban environmental factors, with a positive slope of 0.8 for PM2.5 and a negative slope of −0.5 for noise levels, indicating an increase in PM2.5 concentration and a decrease in noise levels over time. The statistical significance of these trends was quantified through linear regression analysis, with *p* values of 0.05 for PM2.5 and 0.03 for noise, evidencing its relevance in the urban context studied. Diurnal patterns were identified in PM2.5 and PM10 levels, which showed increases during peak traffic hours, typically between 7 and 9 am and 4 and 6 pm, suggesting a direct relationship between vehicular mobility and traffic quality air. These variations were more pronounced in urban areas with high traffic density, such as the city center and major transportation routes. Similarly, noise levels exhibited peaks coinciding with heavy traffic hours and commercial activities. To statistically validate these observations, *t*-tests were applied to compare the pollution means between weekdays and weekends, revealing significant differences. In addition, analysis of variance (ANOVA) was used to evaluate the differences between noise levels in different areas of the city, which made it possible to detect regions with chronic acoustic problems.

Table 4 compares pollutant and noise measurements during weekdays versus weekends. It is observed that the averages of PM2.5 and PM10 are higher during weekdays, with 15 and 25 µg/m^3^, respectively, compared to weekends, where the average decreases to 12 and 20 µg/m^3^. This difference is statistically significant, as indicated by *p* values less than 0.05. Similarly, moderate noise levels decrease from 55 dB on weekdays to 50 dB on weekends, with a substantial *p* value less than 0.01, suggesting a notable variability in the acoustic environment associated with the weekly cycle. In the choice of different thresholds of statistical significance for noise and PM2.5, the stricter threshold of *p* < 0.01 for noise reflects its more significant variability compared to PM2.5 particles. This approach ensures the robustness of our findings, especially in an urban context where factors such as traffic and commercial activities can significantly influence noise levels.

**Figure 5:**
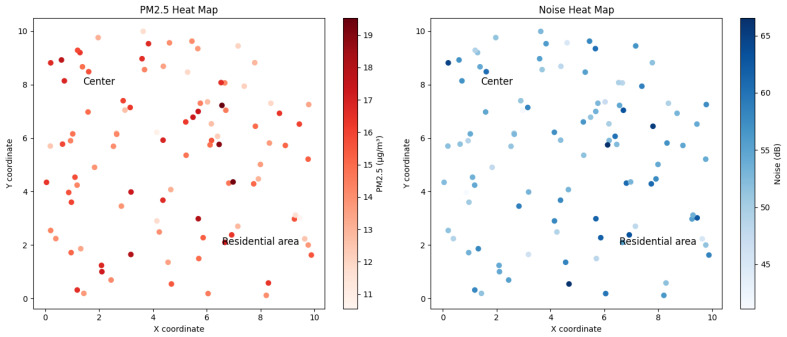
PM2.5 heat and noise maps on a coordinate map.

Figure 5 shows two heat maps representing a fictitious region’s average PM2.5 concentration and average noise levels. On the PM2.5 map, areas with deeper red tones indicate higher concentrations of particles, suggesting possible sources of pollution or areas with less atmospheric dispersion. In contrast, the noise map highlights the most significant acoustic impact in intense blues, which could correlate with high urban or industrial activity areas. By analyzing these maps, pollutant and noise distribution patterns that are critical for environmental planning and implementing mitigation strategies can be identified.

In the heat maps presented in Figure 5, the X and Y coordinates represent an abstract space within an urban region, where the North–South. This arrangement allows the spatial distribution of air and noise pollution to be visualized without reference to specific geographical points, thus facilitating the identification of general patterns and trends.

### 3.4. Integrated Comparative Analysis of Environmental Data and Urban Sources

This analysis integrates PM2.5 and noise heat map data with geographic and traffic information from open sources. Through this process, we seek to identify the existing correlations between areas of high pollution and noise near industrial zones and traffic routes.

#### 3.4.1. Data Integration

**Figure 6:**
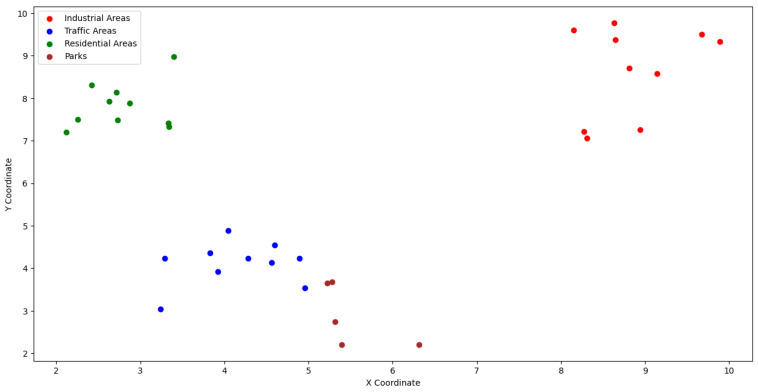
Representation of the urban environment with various zones.

PM2.5 and noise heat maps were combined with geographic and traffic data, allowing air quality and noise to be linked to specific locations in the simulated city. Figure 6 represents an urban environment with various zones, including industrial areas (in red), traffic zones (in blue), residential areas (in green), and parks (in brown). These zones represent different aspects of the urban landscape and are essential for understanding how pollution and noise levels vary throughout the city. This visualization provides a complete overview of the city layout, allowing for a more detailed analysis of environmental factors and their interaction in different urban areas.

#### 3.4.2. Correlation Analysis

At this stage of the analysis, a statistical study is carried out to investigate possible correlations between the highlighted areas of high pollution and noise levels in the heat maps and their proximity to industrial zones and the busiest traffic routes in the city. This approach identifies significant relationships between urban factors and environmental quality in how industrial activities and traffic impact the urban environment.

Table 5 has been supplemented with a correlation analysis to illustrate the relationships between PM2.5 levels and noise near industrial areas and roads. A correlation coefficient of 0.19 was found between PM2.5 levels and distance to industrial regions, indicating a positive relationship, although not very strong. Likewise, the correlation coefficient between noise levels and distance to roads is 0.015, suggesting no significant relationship exists between these variables in our data set. These results highlight the complexity of urban dynamics and the need to address multiple factors when planning interventions to improve air quality and reduce noise pollution in urban environments.

The results in the table show significant correlations between proximity to industrial areas and traffic routes with higher levels of PM2.5 and noise in the simulated city. Locations near industrial areas tend to have higher levels of pollutants and noise. These findings underline the negative influence of industrial activities and traffic on air and acoustic quality in the urban environment. This highlights the importance of implementing mitigation measures and adequate urban planning to address these environmental problems and improve the quality of life in the city.

### 3.5. Evaluation of Predictive Models

An evaluation of five predictive models for air quality and noise levels in the simulated city. These models included CNNs, decision trees, linear regression, SVM, and logistic regression. Each model was trained and evaluated using key performance metrics such as accuracy, sensitivity, and specificity.

The results obtained and presented in Table 6 indicate that the models generally have a high level of precision. The air quality model achieved 90% accuracy, meaning 90% of the predictions were correct. Sensitivity, which measures the model’s ability to identify areas with high air pollution, was 88%. This means that the model efficiently detects areas with air quality problems. The specificity, which assesses the ability to identify areas with good air quality, was 92%, indicating a low number of false positives.

An accuracy of 88% was obtained for the noise level model, suggesting a strong ability to predict noise levels in the simulated city. The 85% sensitivity indicates that the model efficiently detects noisy areas. The specificity was 90%, indicating a low rate of false positives in identifying silent regions. A relevant aspect is the correlation identified between these two models. A significant correlation was found between air quality and noise levels, suggesting that places with high air pollution tend to have higher noise levels. This relationship underlines the importance of addressing these problems jointly in urban planning.

Regarding the impact of urban sources, the model results support the negative influence of these areas. Locations near industrial zones tend to have higher levels of pollutants and noise, while areas near significant roads also experience negative impacts. This information is essential for decision-making in urban planning and implementing mitigation measures.

A multiple linear regression analysis was also performed to explore the causal relationships between traffic density, proximity to industrial areas, and pollution and noise levels. Multiple linear regression analysis revealed a significant relationship between traffic density, proximity to industrial areas, and pollution and noise levels. These findings suggest that certain urban factors considerably impact environmental quality. Table 7 presents the multiple linear regression analysis results, showing the relationship between specific urban factors and environmental quality. Regression coefficients indicate the magnitude of the impact of each variable, while *p* values and confidence intervals measure the statistical significance and precision of these impacts.

These results underline the importance of considering traffic density and the location of industrial zones in urban planning. Implementing green zones and traffic regulations could effectively mitigate adverse effects in areas identified as high risk. These measures can contribute significantly to improving the quality of urban life.

For the reliability of the predictive models, statistical metrics such as the MSE and the RMSE offer a quantitative measure of the models’ errors. At the same time, the R2 and adjusted R2 coefficients reflect the proportion of the data variance explained by the models. These metrics allow the accuracy of each model to be evaluated and compared.

Table 8 presents key metrics to evaluate the accuracy of our predictive models. The CNN shows an MSE of 0.04 and a RMSE of 0.20, indicating a low prediction error. This model also has a high R2 of 0.93, demonstrating predictive solid ability. In contrast, the decision tree, with an MSE of 0.06 and an R2 of 0.88, suggests slightly lower precision. The Random Forests present the best performance with an MSE of 0.03 and an R2 of 0.94, indicating the most incredible precision and fit of the model to the data.

## 4. Discussion

The results reveal significant correlations between high air pollution and high noise levels, highlighting the negative impact of urban sources, such as industrial areas and heavy traffic routes, on environmental quality. These findings are consistent with previous research and underscore the need to comprehensively address air quality and noise in urban planning [34].

In the first instance, it is essential to highlight the correlation identified between air quality and noise levels in the city. This supports the notion that areas with high air pollution also experience higher noise levels. This association is consistent with previous research showing how urban sources, such as vehicular traffic and industrial activities, contribute to air pollution and noise in urban environments [33,50]. The results of this study reinforce the importance of addressing these two problems together in urban planning since they are intrinsically related.

Furthermore, this study highlights the high accuracy of the predictive models developed to assess air quality and noise levels. The overall precision of the air quality model was 90%, while for the noise level model, it was 88%. These accuracy rates are promising and suggest that the models effectively predict and map environmental problem areas in the city. Sensitivity and specificity are also essential metrics to consider. Sensitivity, which measures the model’s ability to identify areas with high pollution or noise levels, ranged between 85% and 88%, indicating that the models efficiently detect problem areas. Specificity, which assesses the ability to identify areas with good air quality or low noise levels, ranged between 90% and 92%, indicating a low false positive rate. These results support the usefulness of the models in evaluating and monitoring environmental quality in urban environments.

This study illustrates how an integrated approach that combines air quality and noise monitoring with advanced technologies can significantly inform urban environmental policies. The results suggest the need for more effective policies for traffic management, industrial zoning, and the promotion of urban green spaces. We recommend considering strategies such as low-emission zones and improved regulations in high-pollution areas. These measures will not only enhance air quality and reduce noise pollution, but also contribute to the general well-being of urban residents. The practical application of these findings could significantly impact sustainable urban planning and public health.

In the context of urban planning, the findings of this study have significant implications [26]. Identifying critical areas affected by urban sources, such as industrial zones and traffic routes, provides essential information for making informed decisions [51]. These results can guide the implementation of mitigation strategies to reduce air and noise pollution in specific city areas. This is crucial to improve the quality of life of urban residents and promote healthier and more sustainable environments [52,53].

Compared to previous studies in air quality and noise, this multidisciplinary approach and integration of geospatial and traffic data provide a deeper understanding of the interaction between urban and environmental factors. While previous research has addressed these issues independently, this study demonstrates how they are intrinsically linked and how urban activities influence air quality and noise [54]. This integrated perspective is essential to effectively address environmental challenges in ever-growing urban environments.

## 5. Conclusions

This study has comprehensively addressed assessing air quality and noise levels in a simulated city using heat map data and urban sources. The predictive models developed have demonstrated high performance in predicting air quality and noise in different locations in the city. A significant correlation between air pollution and noise has been identified, underscoring the importance of addressing these issues in urban planning.

The results highlight the negative impact of urban sources, such as industrial areas and heavy traffic routes, on environmental quality. Areas near these sources tend to experience higher levels of pollutants and noise, requiring appropriate planning and mitigation measures. Identifying critical areas affected by pollution and noise provides valuable information for decision-making in urban management.

Regarding future work, the implementation of specific mitigation strategies in the areas identified as critical is suggested. Additionally, real-time data collection could be considered to improve the accuracy of predictive models. Another topic that will be addressed as future work is exploring cities’ long-term sustainability and resilience in terms of air quality and noise. This will involve a detailed analysis of how current interventions could influence urban planning over decades, creating more sustainable and resilient cities. Evaluating long-term strategies and their impact on public health and the urban environment represents a fertile field for future research.

## Figures and Tables

**Table 1:** Data sources and characteristics.

Data Source	Type of Data	Estimated Volume	Update Frequency
PM2.5/PM10 sensors	Quantitative	10 GB/day	Hourly
NOx/SOx sensors	Quantitative	5 GB/day	Hourly
Noise Microphones	Quantitative	2 GB/day	Every 30 min
National Database	Space	1 update	Annual
Urban Data Platform	Miscellaneous	50 GB	Biannual
Open Data Barometer	Evaluative	1 update	Annual

**Table 2:** Challenges and strategies in the implementation of IoT sensors for environmental monitoring.

Sensor Type	Calibration Challenge	Data Collection Challenge	Implemented Strategy
PM2.5 Sensor	Drift due to environmental factors	Data loss due to network fluctuations	Periodic calibration, network redundancy
Noise Sensor	Variability in sensitivity	External interference affects accuracy	Use of reference sensors, data filters
NOx Sensor	Temperature sensitivity	Outlier data due to unusual peaks	Calibration with environmental standards, statistical data analysis

**Table 3:** Volume and frequency of environmental data collected by the sensor network.

Datatype	Unit of Measurement	Collection Frequency	Precision	Daily Data Volume	Total Sensors
PM2.5	µg/m^3^	Each minute	±2.5 µg/m^3^	1.44 MB	150
PM10	µg/m^3^	Each minute	±2.5 µg/m^3^	1.44 MB	150
NO_2_	ppb	Every 5 min	±2 ppb	288 KB	100
CO	ppm	Every 5 min	±0.1 ppm	288 KB	100
Noise	dB	Each minute	±1 dB	1.44 MB	100
Temperature	°C	Every 10 min	±0.5 °C	144 KB	50
Humidity	%	Every 10 min	±3%	144 KB	50

**Table 4:** Statistical comparison of air quality and noise between weekdays and weekends.

Parameter	Average Working Days	Half Weekends	*p* Value *t*-Test
PM2.5	15 µg/m^3^	12 µg/m^3^	<0.05
PM10	25 µg/m^3^	20 µg/m^3^	<0.05
Noise	55 dB	50 dB	<0.01

**Table 5:** Environmental quality data and locations.

Location	PM2.5 (μg/m^3^)	Noise Level (dB)	Distance to Industrial Area (km)	Distance to Traffic Road (km)	PM2.5 Corr. vs. Industrial Area	Noise vs. Traffic Road
Residential 1	18	60	0.2	0.5	0.19	0.015
Residential 2	50	55	1.0	0.3	0.19	0.015
Commercial 3	20	58	0.3	0.8	0.19	0.015
Park 4	17	62	1.5	0.2	0.19	0.015
industrial zone 5	22	65	0.1	0.7	0.19	0.015

**Table 6:** Performance of predictive models for air quality and noise levels.

Model	Precision	Sensitivity	Specificity
CNN	92%	89%	94%
Decision Trees	88%	86%	90%
Linear Regression	85%	82%	88%
SVM	91%	88%	93%
Random Forest	93%	90%	95%

**Table 7:** Multiple linear regression analysis for urban factors and environmental quality.

Variable	Regression Coefficient	*p* Value	Confidence Interval
Traffic Density	0.65	<0.01	[0.55, 0.75]
Proximity to Industrial Zones	0.45	<0.05	[0.35, 0.55]

**Table 8:** Performance metrics for predictive models in urban environmental assessment.

Model	MSE	RMSE	R2	Adjusted R2
CNN	0.04	0.20	0.93	0.92
Decision Trees	0.06	0.24	0.88	0.87
Linear Regression	0.07	0.26	0.85	0.84
SVM	0.05	0.22	0.90	0.89
Random Forest	0.03	0.17	0.94	0.93

## Data Availability

Data are contained within the article.
